# Statistical Data Analysis on the Trends of Time Series Activities: Potential Correlation between Computer-based Activity and Health Lifestyle Activity

**Published:** 2018-09

**Authors:** Mehdi REZAEI, Myoungsug CHUNG, Taikyeong JEONG

**Affiliations:** 1. Dept. of Forestry and Landscape Architecture, Konkuk University, Seoul, Republic of Korea; 2. Dept. of Industrial Engineering, Ajou University, Suwon, Republic of Korea; 3. College of Business, CHA University, Pocheon-si, Republic of Korea

**Keywords:** Computer-based activities, Healthy lifestyle, Time series activities, Leisure time activity, Korea

## Abstract

**Background:**

We aimed to investigate the trends of interested and extra time spending activities in order to find potential correlation between computer-based activities (CBA) and healthy lifestyle (HLA).

**Methods:**

Information was adapted from the South Korea governmental open source database which gathered from official measurement statistical results. Various types of the interested and extra time spending activities were categorized into eight main activities based on the library research and expert comments. Moreover, two main categories of sports and outdoor activity (S&OA) were found to be attributed to HLA. Descriptive and analytical statistics analyses, besides correlation analysis were conducted; through Kolmogorov-Smirnov test for normalizing data and Fisher’s exact test for making the comparison.

**Results:**

Among demographic variables, watching and listening as well as social activities, were the most interesting activities for almost all citizens, furthermore computer games and social network system (CG&SNS) were found to have a negative association with HLA.

**Conclusion:**

Newly emerged computer-based activities, such as game behavior, would be among the main determinates of the HLA. The associated implications are provided to assist the authorities and governments in making the policy and planning.

## Introduction

Several countries around the world are going to be more and more attached to the technology, and apply it in various perspectives of citizens’ daily lifestyle. This phenomenon had sparked a debate about its positive outcomes and drawbacks, due to its potential to either provide a more convenient lifestyle (1) or leading people to some associated disease (2), respectively. In fact, the level of physical activities is less than past because of the more computer-based lifestyle (3), while it is still a necessity for the human to have a healthy lifestyle (2). Furthermore, in a larger scale, poor physical activity is the cause of some diseases such as coronary heart disease, strokes, high blood pressure, breathlessness and overweight (2), which indicates on negative impacts for individuals, families and the government.

We aimed to investigate among the citizens of South Korea, as a high tech. country (4). This country has become the seventh largest advanced economy in the world (5) and is experiencing a sustainable development to improve the lives of the people (6). What made this country unique in this regard is that the change has happened so fast which might cause several impacts on the lifestyle of the citizens; for instance, making a significant change on their attitude to spend their time activity, and accordingly healthy lifestyle (HLA).

Nowadays, expanding computer games and games behavior among the people’ leisure and extra time is a big concern for governments, at large; because it has a direct impact on the community’s health and behavior. This pattern is a crucial component of a balanced HLA. Physical activity and sports have been claimed to play a pivotal role in health prevention policies (7), so recognizing the leisure time activity (LTA) trend is likely to be noticeable to find the HLA trend in a society and accordingly to reach the implications for the planning. More studies have investigated the impact of the computer-based activities on the HLA of the Korean residents (8, 9).

We aimed to analyze the trend of leisure time activities (LTA) among the Korean citizens, in order to find the potential correlations between the impacts of computer-based activities and HLA. The results can contribute to clarifying the trend of game behavior and its corresponding impacts on a community.

## Methods

### Data collection

The data were adopted from the open data source provided by the governmental agencies in South Korea from 2015 to 2016 (10). All data were ingested based on four criteria: namely, age, gender, income, and occupation. After the process of data interoperation, we obtained the final categories and classifications.

### Classification of Leisure and Extra time activity

**Fig. 1:**
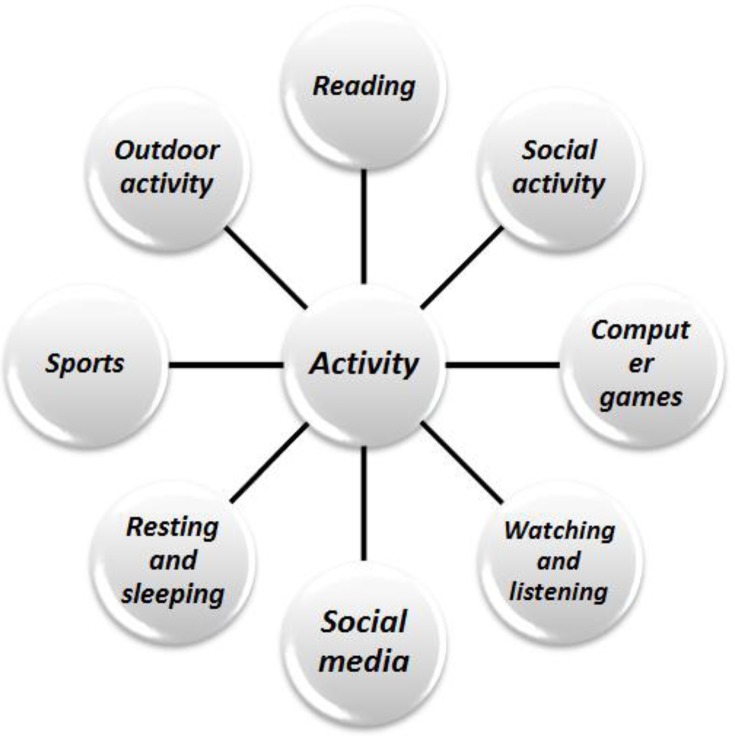
Physical activities classification based on the export comments

In most of the scientific research, leisure time activity (LTA) is defined as a quality of experience or as free time (11, 12). It is the time, spent away from the business, work, education and involves the activity that people are interested in to do (13). The variable of LTA can be quantified, measured and compared (14). We classified 118 activities, such as Internet Games, Play stations, watch TV, online chatting, football, mountain climbing, walking, meetings with friends, aerobic, taekwondo, participation in the local festival, volleyball, etc. in eight main activities based on the library research and expert comments (Fig. 1).

### Indicators of a healthy lifestyle

HLA has a broad definition; we used the definition provided by WHO; it is claimed both physical and mental health are involved. Not only does it refer to a ‘way of living that lowers the risk’, but also it involves physical, mental and social well-being (2) and it will cause corresponding impacts on the family and society. Moreover, they suggested five main factors that determine a healthy lifestyle namely, ‘Tobacco, Physical Activity, Healthy Eating, and Alcohol’ (2).

Therefore, we recognized the trend of different interests on the physical activities that play an important role in health and wellbeing of a community. Hence, sports and outdoor activities (S&OA) were considered as the indicators of HLA.

### Data analyses

The most obvious trends on the data were examined in terms of game HLA and computer-based activities. Firstly, the trends of LTA interests among the participants were analyzed based on their demographic information, namely, gender, age, occupation and income. Each category of the measured activities was computed from the mean of several other associated activities, the process was explained in Fig. 1, in which each category was emerged based on several other associated sub-categories. The normality of the data (15, 16) also was tested using descriptive statistics and Kolmogorov-Smirnov test in the SPSS. This comparison is quite well fit into our data and correlation ship investigation because the data had similar statistical distribution and it is a proximate test easily computed in any dimension (17, 18).

In addition, we investigated the general situation of HLA. The data analysis involved descriptive and analytical statistics. The correlation between LTA and HLA were conducted using Charts analysis. Moreover, in order to ensure the accuracy of the results, Fisher’s exact test was conducted for each category since it is most accountable in contingency tables (19–21).

## Results

### Analyzing the trend of leisure time activity based on gender

**Fig. 2:**
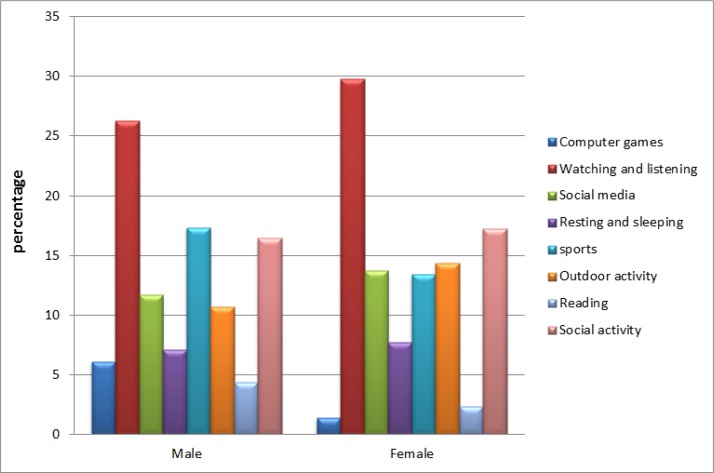
Trend of Interests on leisure time activates based on gender

Fig. 2 provides the distribution of interests on LTA based on gender. ’Watching and listening’ is the most interesting activity for both genders, it is followed by social activity, and sport for female and male, respectively. Sports is more interested in male, while, female prefer the outdoor activity. Regarding the computer games, the rates for male is almost 3 times more than that of female.

Comparing the differences based on the statistical analysis revealed that there were no significant differences for male and female in general situation (*P*<0.05). Nevertheless, presenting the information visually, mere differences can be seen. Accordingly, although the attitude to use computer game is, comparatively, higher for male, they are likely to have a healthier lifestyle due to their higher tendency to sport and outdoor activities. However, the rates are almost similar. The results of HLA analysis, showed the rates of 28.5 and 27.75 for male and female, respectively.

### Analyzing the trend of leisure time activity based on age

**Fig. 3:**
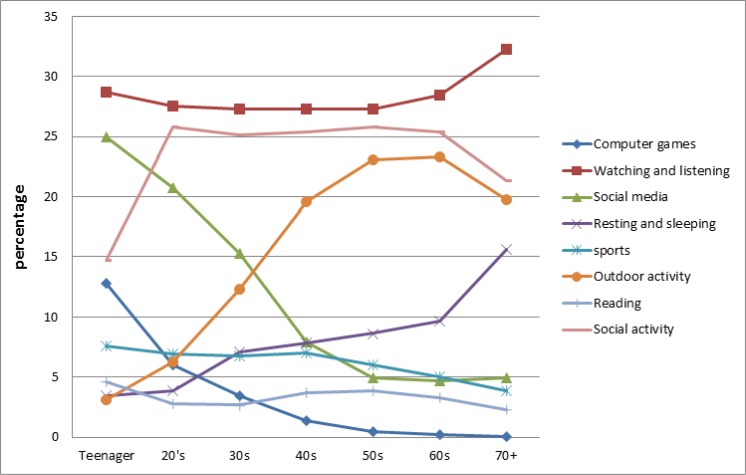
Trend of Interests on leisure time activates based on age

As presented in Fig. 3, watching and listening as well as social activities were the most preferred activities among all groups of ages, while reading was the least welcomed activity.

There is a positive correlation between the age and interests in outdoor activities; in contrast, this correlation is negative for the interests to use social media. Likewise, the interest to use computer game is dramatically higher than that of older individuals, and a sharp decreasing trend can be seen among teenagers to people in their 40 decades.

This interest is almost zero for people older than 50. Regarding sports, there is a slight downward trend as participants were older.

Two groups of teenagers and people in their 20s have the highest rate of interests for both computer game and social network/media (CG&SNS). Moreover, the trends of interests in different type of activities, apparently, differ based on the age.

**Fig. 4:**
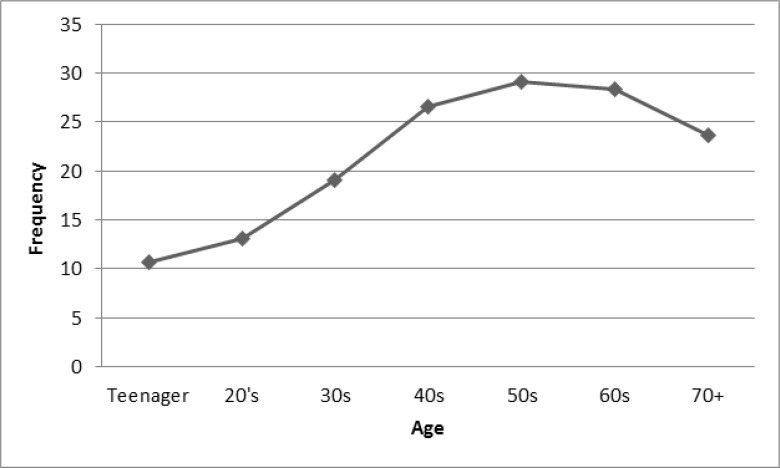
Trend of HLA based on age

Analyzing Fig. 4, developed based on the HLA associated factors, revealed that people in their 50’s are on top of the graph, and it is followed by 60’s and 40’s aged individual. The lowest rate is for people aged 20’s and younger, which seem to be logical based on their higher interests in CG&SNS.

### Analyzing the trend of leisure time activity based on occupation

**Fig. 5:**
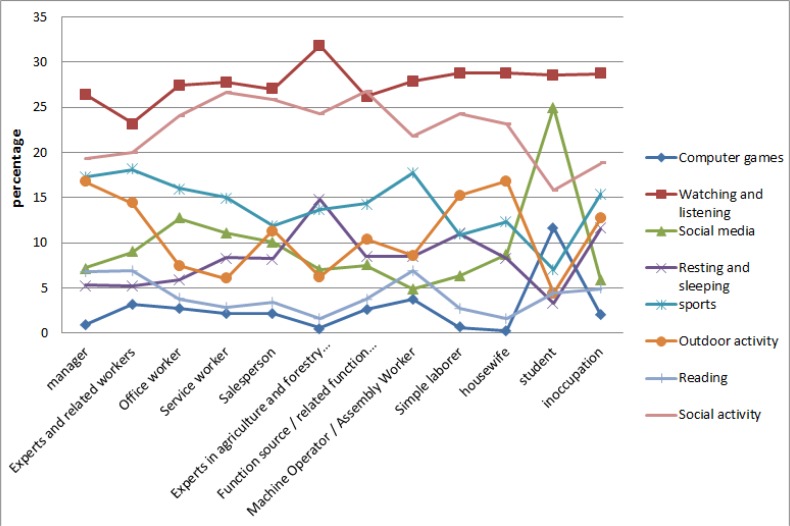
Trend of Interests on leisure time activates based on occupation

Watching as well as social activity is the most interested LTA among all occupations, while the least interesting activity was computer game (Fig. 5).

However, this trend is completely different for students. The other interests among the students also differ from the trends in other occupations, which implies on their age group as well. For instance, outdoor, social activity and sleeping are the lowest interesting activities for students, while, social media and computer games are the highest.

**Fig. 6:**
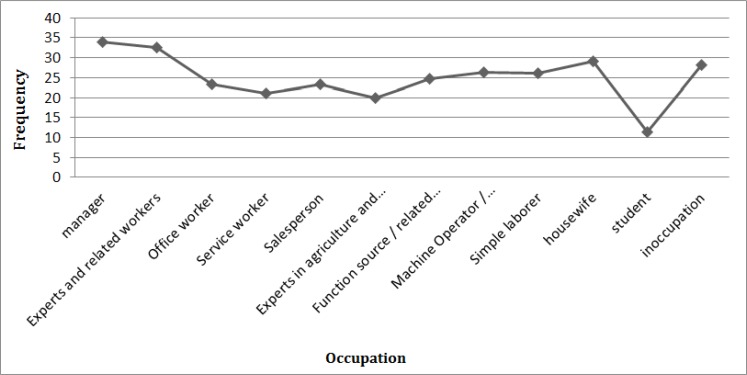
Trend of HLA based on occupation

According to the HLA factors (Fig. 6), managers are likely to have the healthiest activities comparing with other occupations, and it is followed by participants classified as expert, with 34.02% and 32.51%, respectively. Although other accusations are likely to have quite similar interests in different LTA, these rates seem to be different for students.

### Analyzing the trend of leisure time activity based on income

**Fig. 7:**
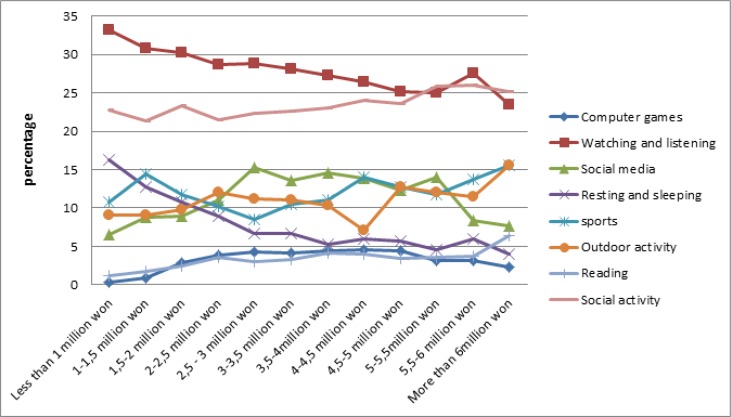
Trend of Interests on leisure time activates based on income

Similar to other trends, watching and social activities are the most interested LTA for people at different levels of income. However, the lowest interested LTA were computer game and reading. Analyzing the data from the perspective of income, no significant differences were shown. The major differences were for the outdoor activity, which has a nearly negative correlation with income; nevertheless, a noticeable decline of interest is for people with the 4 to 4.5 million won (KRW) income, approximately 3.5K USD. There is a negative correlation between the income and people’s interest in resting (Fig. 7).

**Fig. 8:**
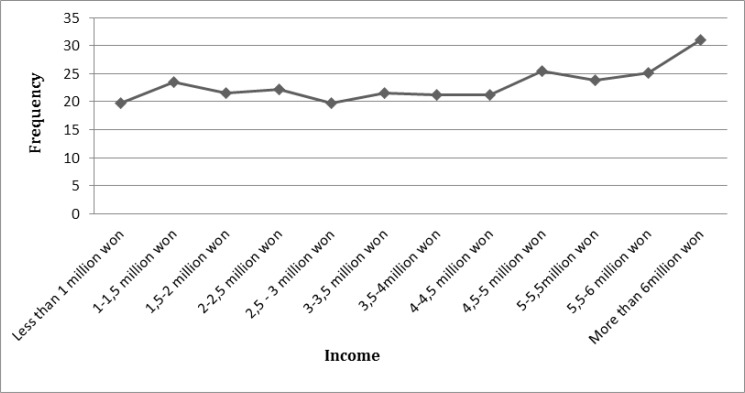
Trend of HLA based on income

There is a negative correlation between the amount of income and the interests to have a healthy lifestyle. However, the factor of income does not lead to significant changes, except for people with the income of more than 6 million won. Furthermore, computer game is the most interested among people with the average amount of income, 2.5 to 5 million won (Fig. 8).

## Discussion

The purpose of this study was to analyze the trend of people’s different activities in spending their time and its impact on HLA in a computer-based community. Accordingly, the general trend of HLA in a community could be assessed based on the citizens’ interests in health-related activities or, newly emerged, computer-related one, particularly game behavior.

Watching and listening as well as social activities, were the most interested activities for almost all citizens, regardless their demographic variables; meanwhile, the rates of healthy relevant outdoor activities (S&OA) and computer-based activities (CG&SNS), differ based on each variable.

Considering the age, elderly people are likely to have a healthier life owing to higher rates of exercise and lower rate of computer-based activities. The HLA factor for middle-aged people (approximately 30–50 yr old) was about 3 times more than that of teenagers. The younger generation has a higher tendency to the CG&SNS rather than S&OA. The innovations of technology have changed the healthy lifestyle among different generations in South Korea. Similarly, computer game, TV and social network or media cause an unhealthy lifestyle (22). Moreover, male and female were quite similar and not a logical contribution could be found, though, the rate of HLA was slightly higher for male.

Analyzing the occupation has revealed that more prestigious jobs seem to provide a healthier lifestyle for its employees, though only physical activities were included here and the importance of other factors, such as level of stress at work, organizational satisfaction or other mental diseases (23) should be taken into account as indicators. Meanwhile, younger generation, categorized as students, prefer CG&SNS as the most attractive activities.

Besides, the impact of occupation, the level of income might also play a role in changing the tendency of people to have healthier life. CG&SNS was more welcomed by people with median level of income.

Accordingly, there is a negative correlation between the tendency to the CG&SNS and having an HLA. Not only does it lead to a healthier life, but also quality of life is highly correlated to spending time with physical activities (7), rather than computer-based one. The more computer-based activities, the less HLA will be. Such trend could be attributed to several factors, such as gender, age, occupation, and income. There are several other indicators which might play a role, for instance, specific barriers to having outdoor activities previously mentioned in the case of South Korea (24–26).

Authorities and governmental policy could provide not only more educational programs (9), but also facilities and supports can be improved in order to enable the citizens to have a healthier lifestyle. It is mostly required for the younger generation who are more interested in game and computers, rather than healthy activities.

## Conclusion

The newly emerged interests and extra time spending activities on computer-based activities seem to play a pivotal role in the healthy lifestyle of a community. Although a specific group of indicators was tested in this research, we assumed outdoor activities and sports as the main determinants of an HLA. Meanwhile, there are several other factors that might play a role covered by further studies and from different perspectives.

## Ethical considerations

Ethical issues (Including plagiarism, informed consent, misconduct, data fabrication and/or falsification, double publication and/or submission, redundancy, etc.) have been completely observed by the authors.
